# Miscellany

**Published:** 1894-01

**Authors:** 


### Miscellany.

### SURGICAL SUGGESTIONS.

(*Medical Record, December, 1893.*)

SUPERFICIAL EXCORIATION produced by braces or adhesive plaster are readily healed by the free application of the sub-iodide of bismuth. It is a fine red powder, with unusual absorbent and antiseptic powers.

ACUTE hydrocele in children, if tapped with a small trocar and cannula, can almost invariably be cured if the sac is irritated, after the evacuation, with the blunt end of the cannula.

POTASSIUM permanganate solution (1 to 1000) is an excellent local antiseptic for the urinary tract. One of the advantages it has over other agents is, that so long as pus or other excrementitious matter is present, the fluid will be changed in color from the deoxidation. For acute gonorrhea it is particularly applicable, and in the preparation of cases for operation.

MULTIPLE OPERATION AT ONE SITTING.—For the past two years, I have frequently done several operations at one sitting. I have repeatedly repaired the cervix and perineum and curetted the cavity of the uterus before opening the abdomen for the removal of pus tubes, and afterward stitched the fundus of the uterus to the anterior abdominal wall with silkworm-gut, and have, thus far, seen no cause to regret doing so.—*Cushing.*

Be prepared to go ahead the moment patient is unconscious; don't waste either the anesthetic or the patient's forces while you are threading needles.

Find the source of hemorrhage in gunshot wounds of the abdomen before you begin sewing up the wounds in the intestines. —*Dalton.*

Gall-stones call for operation when they cause frequent, repeated or long-continued trouble. Operation is required for empyema of gall bladder, and for hydrops too, if it causes much annoyance. If cystic duct is closed, gall bladder inflamed, or its contents much altered, a temporary biliary fistula should be made.—*Czerny.*

Gastrostomy.—In the treatment of impermeable cicatricial stenosis of the esophagus, gastrostomy not only furnishes a new inlet for the introduction of food into the stomach, and thus prevents death from starvation, but it often proves a curative measure in such cases, as the gastric fistula can be utilized for another purpose—successful retrograde dilatation of the stricture.—*Senn.*

---

Dr. Benjamin H. Grove desires to announce the change of his residence—but not of office—to 101 Jewett avenue. His new offices are virtually located as before—now, however, in the main portion of the building—at No. 334 Pearl street, near Huron street, opposite the Morgan building. Hours, 9 a. m. to 1 p. m.; Sundays, 1 to 2 p. m.; also, Wednesday and Friday evenings, 6.30 to 7.30.

---

The American Medical Publishers' Association held its first annual meeting in the Grand Hotel, Cincinnati, on Monday, December 4, 1893, and steps were taken in the direction of active routine work. The by-laws and rules were revised and amended, while the name was modified in accordance with a demand from medical publishers of a general nature who desired to become members of the Association. The active coöperation of every medical publisher is earnestly solicited. Next meeting in Washington, D. C.,

September, 1894. Officers: President, Dr. Landon B. Edwards, Richmond, Va.; Vice-President, Dr. J. C. Culbertson, Cincinnati, Ohio; Treasurer, J. MacDonald, Jr., New York City. For application blanks and copies of the articles of association, address Charles Wood Fassett, Secretary, corner Sixth and Charles streets, St. Joseph, Mo.

---

The firm of Hummel & Parmele (Medical Journal Advertising) was formed by Dr. A. L. Hummel and Charles Roome Parmele, under articles of partnership, which expire by limitation December 31, 1893.

In March, 1892, Mr. Parmele became secretary and treasurer of The Papoid Co., and sold to Dr. Hummel his interest, good will, etc., in the firm of Hummel & Parmele. Since March, 1892, Mr. Parmele has devoted his entire time and attention to The Papoid Co., and Dr. Hummel has conducted the business of Hummel & Parmele.

After the expiration of the articles of partnership on December 31, 1893, the Medical Journal Advertising business of Hummel & Parmele will be carried on by Dr. Hummel, as heretofore, under the firm name A. L. Hummel, M. D., Medical Journal Advertising, at 257 South Fourth street, Philadelphia, Pa.

---

Bits of Information.—An excellent hair tonic is made by scalding two ounces of black tea in a gallon of boiling water; strain, and add three ounces of glycerine, tincture of cantharides, one-half ounce, and bay rum, one quart. Mix well by shaking, and then perfume.

When massaging the face, rub lines under the eyes, from the nose to the temples. This is the rule. In washing the eyes, wipe them from the temples to the nose. This is said to prolong sight.

If you wish to have a sweet breath, use a tooth powder which contains camphor.

Sponge bathing with alcohol is excellent for delicate women.

A simple remedy for a rough skin is to first wash the face thoroughly at night, then rub it with about a teaspoonful of cream, and let it dry in. The skin will look shiny and feel stiff at first; but in the morning you will be surprised to find how soft the skin will be.—*Reflector.*

Some Reasons for Daily Exercise.—1. Any man who does not take time for exercise will probably have to make time to be ill.

2. Body and mind are both gifts; and for the proper use of them our Maker will hold us responsible.

3. Exercise gradually increases the physical powers, and gives more strength to resist sickness.

4. Exercise will do for your body what intellectual training will do for your mind—educate and strengthen it.

5. Plato called a man lame, because he exercised the mind while the body was allowed to suffer.

6. A sound body lies at the foundation of all that goes to make life a success. Exercise will help to give it.

7. Exercise will help a young man to lead a chaste life.

8. Varied, light, and brisk exercises, next to sleep, will rest the tired brain better than anything else.

9. Metal will rust if not used, and the body will become diseased if not exercised.

10. A man "too busy" to take care of his health is like a workman too busy to sharpen his tools.—*Reflector.*

